# Geometric Distribution-Based Readers Scheduling Optimization Algorithm Using Artificial Immune System

**DOI:** 10.3390/s16111924

**Published:** 2016-11-16

**Authors:** Litian Duan, Zizhong John Wang, Fu Duan

**Affiliations:** 1 College of Information Engineering, Taiyuan University of Technology, Taiyuan 030024, China; duanlitian0030@link.tyut.edu.cn; 2 Department of Mathematics and Computer Science, Virginia Wesleyan College, Norfolk, VA 23502, USA; zwang@vwc.edu; 3 College of Computer Science and Technology, Taiyuan University of Technology, Taiyuan 030024, China

**Keywords:** multiple-reader environment, multiple-reader interference, geometric distribution probability function, optimization by artificial immune system

## Abstract

In the multiple-reader environment (MRE) of radio frequency identification (RFID) system, multiple readers are often scheduled to interrogate the randomized tags via operating at different time slots or frequency channels to decrease the signal interferences. Based on this, a Geometric Distribution-based Multiple-reader Scheduling Optimization Algorithm using Artificial Immune System (GD-MRSOA-AIS) is proposed to fairly and optimally schedule the readers operating from the viewpoint of resource allocations. GD-MRSOA-AIS is composed of two parts, where a geometric distribution function combined with the fairness consideration is first introduced to generate the feasible scheduling schemes for reader operation. After that, artificial immune system (including immune clone, immune mutation and immune suppression) quickly optimize these feasible ones as the optimal scheduling scheme to ensure that readers are fairly operating with larger effective interrogation range and lower interferences. Compared with the state-of-the-art algorithm, the simulation results indicate that GD-MRSOA-AIS could efficiently schedules the multiple readers operating with a fairer resource allocation scheme, performing in larger effective interrogation range.

## 1. Introduction

Radio Frequency Identification (RFID) is one of the Auto Identification and Data Capture (AIDC) techniques, which enable objects to be automatically identified. With the advantages such as automation, no-contract, easy-to-implement and enough-durability, passive RFID is widely applied in various applications such as supply chain management [1], manufacturing management [2] and warehouse management [3] with objects localization [4].

According to most applications, a single reader cannot totally cover a specific area (i.e., in a warehouse, every product has a tag attached to by automatically taking stock). Correspondingly, a classical RFID scenario is usually composed of multiple readers and large quantity of passive tags: the so-called Multiple-reader Environment (MRE). In classical MRE, multiple readers are deployed in different densities with known positions to interrogate the randomized tags with fixed positions under the same RFID system. To dynamically allocate the operating resources (i.e., available time slots and frequency channels) and manage readers, centralized mechanism is usually designed to be executed by a coordinator device (i.e., polling server), which is connected to the readers through a wired or wireless network [5,6]. During this process, signal interference problem degrades the operating performance in efficiency and reliability, which is caused by multiple readers operating at the same time. Correspondingly, the multiple-reader interference avoidance algorithms are proposed to help coordinator device to schedule the readers operating non-simultaneously or simultaneously but at different frequency channels, which could be clustered in two categories: the control-mechanism-based ones and the scheduling-based ones.

Control-mechanism-based algorithms [7,8,9] allow each reader to detect the status of other readers in order to avoid the simultaneous operating. For example, Neighbor Friendly Reader Interference Avoidance Algorithm (NFRIA) utilized readers with two bistatic antennas, where one antenna is for data transmitting and the other one is for continuously receiving to detect if other readers are using the same channel when they are operating. However, NFRIA did not take full advantage of centralized mechanism and, meanwhile, it is based on a hardware assumption that makes it hard to implement in real MRE.

Scheduling-based algorithms transform the multiple-reader interference problem into an optimization problem with respect to the allocation of operating resources (e.g., [10,11]). Based on this, the number of time slots, the number of frequency channels and the position of readers are optimally allocated by using some heuristic algorithms such as genetic algorithm [12], swarm optimization algorithm [13] and artificial immune algorithms [14,15,16,17]. Among these, the artificial immune algorithms output the best performance when compared with other heuristic ones, the advantage of which is mainly embodied in outputting a larger interrogation range. In [14,15,16,17], the author introduced a reader-to-reader avoidance model formulizing from the signal-to-interference-plus-noise (SINR) view, which could perfectly describe the interference process. However, the author did not consider the non-uniformity distribution of multiple readers deployment in MRE, resulting in the unfairness of scheduling schemes since the readers with no interferences are always operating while the ones dramatically affected by interferences are almost never operating. Therefore, part of the interrogation range is actually thriftless and, meanwhile, the interfering readers are not effectively scheduled to operate as well.

In the view of scheduling-based mechanism, a Geometric Distribution-based Multiple-reader Scheduling Optimization Algorithm using Artificial Immune System (GD-MRSOA-AIS) is proposed as a resource allocation algorithm to fairly schedule the readers operating with low interferences and large effective interrogation ranges in centralized MRE. GD-MRSOA-AIS first produces several fair feasible scheduling schemes using geometric probability distribution to allocate the operating time slot and frequency channel, which could minimize the collisions among the readers contending for operating resources and therefore maximize the probability that one reader successfully gains the opportunity to operate. To optimize these feasible scheduling schemes with the objective of maximizing the total effective interrogation range, the artificial immune system optimization with immune clone, immune mutation and immune suppression is further introduced. During the mutation process in artificial immune system optimization, there are two alternative mutation operators for different cloned antibodies, where the single-bit mutation is suitable for the superior ones and the region-bits mutation is appropriate for the regular ones.

Summarizing, the contributions of this paper are as follows:

- By using signal-to-interference-plus-noise (SINR) to explain the reduction in interrogation range, a resource allocation model is constructed with two constraint conditions which are used to decrease the multiple-reader interference.
- A geometric probability function is introduced to replace the uniform probability function to produce the feasible scheduling schemes, which ensures that readers could operate in a fair way.
- Artificial immune system with clone operator, mutation operator and suppression operator is introduced to optimize the feasible scheduling schemes. In mutation process, both of the single-bit mutation operator and the region-bits mutation operator are imported to deal with the different cloned antibodies to accelerate the optimization process and convergence speed.

The remainder of this paper is organized as follows. In Section 2, the formulized resource allocation model is constructed in the view of signal-to-interference-plus-noise (SINR) to efficiently avoid the multiple-reader interference in MRE. The structure of GD-MRSOA-AIS is introduced in details in Section 3, where a geometric distribution probability is first utilized to generate the feasible scheduling schemes and then an artificial immune system is designed to optimize these feasible scheduling schemes as optimal ones. Simulation results for GD-MRSOA-AIS are provided in Section 4 as well as a series of comparative results between the proposed one and the state-of-the-art algorithm (i.e., LIs [14]). Finally, the conclusions and future works are drawn in Section 5.

## 2. Resource Allocation Model in Centralized MRE under EPCGlobal C1G2

### 2.1. General Description with Assumptions

In MRE, each reader transmits electromagnetic wave to create the interrogation range, where the size and shape of interrogation range depends on many factors [5,18] such as the antennas design (radiation pattern, gain, polarization and impedance), tag parameters (e.g., gain, matching features and IC sensitivity), external factors (ambient conditions, and noise) and readers output power suited. In most literatures [5,14,15,16,17,19], the interrogation range is defined as a perfect circumstance where the radius only depends on the reader’s output power. In this paper, we also follow this assumption as UHF RFID readers with known positions and passive RFID tags with fixed positions in centralized MRE:

- Readers are randomly distributed over an area in two dimensions.
- Any reader has only two states, active and inactive, where only the readers in active operate and interfere with other active ones.
- The interference power from multiple interfering readers seen by the desired reader is additive.

### 2.2. Multiple-Reader Interference Problem in Centralized MRE

**Figure 1:**
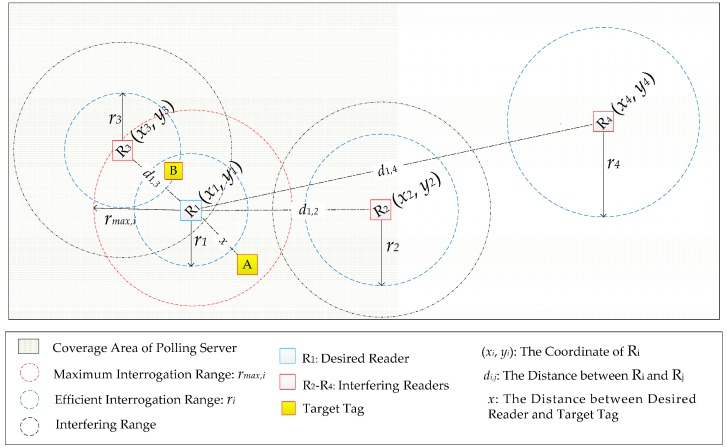
MRE Illustration with Centralized Mechanism.

A simple centralized MRE is illustrated as Figure 1 according to the above assumptions, which is composed of one polling server, four readers in the same rated power and two target tags. Suppose that R_1_ is the desired reader, R_1_ could reach to the red circle as its maximum interrogation radius of *r_max,_*_1_ without any interference. When R_1_ attempts to detect the target tag A, if the signal is masked by the interfering reader R_2_ and R_3_ (the interfering range is represented by the black circle), the effective interrogation range r*_1_* (blue circle) of R_1_ will decrease from *r_max,_*_1_. If *r*_1_ is smaller than the distance *x* between R_1_ and tag A, R_1_ could not detect this target tag A. Moreover, target tag B located in the overlapped range of *r*_1_ and *r*_3_ is hardly detected due to the multiple electromagnetic waves from R_1_ and R_3_ simultaneously operating.

The maximum interrogation radius *r_max,i_* of R*_i_* is related to the hardware design [20], which is obtained by Equation (1), where *λ_i_* is the wavelength of R*_i_*, *P_min_* is the minimum power required for the tag to operate, *P_i_* is the signal transmission power of R*_i_*, *M_d_* is the modulation depth, and *G_T_* and *G_R_* are the gains of the transmit antenna and the receive antenna, respectively.

(1) $$r_{max,i}=\left(\frac{\lambda_{i}}{4\pi }\right)\sqrt{\frac{G_{T}G_{R}P_{i}}{P_{min}}\times \frac{1-M_{d}{}^{4}}{{\left(1+M_{d}\right)}^{2}}}$$

However, the effective interrogation radius r*_i_* of R*_i_* is variable as a function of signal interfering. For reader R*_i_* interrogating the target tag at a given time slot (i.e., at the *k*th time slot), R*_i_* receives the backscatter signal from the tag and, meanwhile, it is also interfered by other simultaneous operating readers signal among *N_Reader_* readers, where a 0–1 function *ω_j_*(*k*) (as Equation (3)) is to indicate if the reader R*_j_* is operating at the *k*th slot or not. To ensure that R*_i_* could successfully detect the target tag in distance of *x,* Signal-to-Interference-Plus-Noise ratio (*SINR*) [21] has to exceed the minimum one of *SINR_min_* as Equation (2):

(2) $$SINR_{i}(x,d_{j,i})=\frac{BP_{i}(x)}{\sum_{j=1\&j\neq i}^{N_{Reader}}\omega_{j}(k)I_{j}(d_{j,i})+No_{i}}\geq SINR_{min}$$

(3) $$\omega_{j}(k)=\{\begin{array}{ll} 1, & R_{j}\;\mathrm{operates}\;\mathrm{at}\;k\mathrm{th}\;\mathrm{slot}\\ 0, & \mathrm{Otherwise} \end{array}$$

where *No_i_* represents the noise power of R*_i_*, *BP_i_*(*x*) represents backscatter signal power from the target tag as a function of the distance *x* between the desired reader and the target tag, *I_j_*(*d_j,i_*) represents the interference from any other R*_j_* as a function of the relative distance *d_i,j_* (as Equation (6)) between the desired reader R*_i_* and the other interfering reader R*_j_*. The function [19] of *BP_i_*(*x*) and *I_j_*(*d_j,i_*) are defined as Equations (4) and (5), respectively.

(4) $$BP_{i}(x)=\alpha_{bw}E_{tag}P_{i}G_{T}G_{R}{\left(P_{0}x^{-\gamma }\right)}^{2}$$

(5) $$\begin{array}{l} I_{j}(d_{j,i})=hP_{j}G_{T}G_{R}\beta_{mask}(\Delta CH_{j,i}(k))P_{0}d_{j,i}^{-\gamma },\\ \Delta CH_{j,i}(k)=|CH_{i}(k)-CH_{j}(k)| \end{array}$$

(6) $$d_{j,i}=\sqrt{{\left(x_{i}-x_{j}\right)}^{2}+{\left(y_{i}-y_{j}\right)}^{2}}$$

where *α_bw_* is the normalized spectrum power, *E_tag_* is the effective power reflection coefficient of the tag, *P_i_* denotes the signal transmission power of R*_i_*, *G_T_* and *G_R_* are the gains of the transmit antenna and the receive antenna, respectively, *P*_0_ is the referenced path loss at the distance of 1 m, *γ* is the path loss exponent, *x* is the distance between R*_i_* and the target tag, *h* is a fading coefficient in the channel between R*_i_* and R*_j_*, *β_mask_*(·) is the function of a spectrum mask defined in EPCGlobal Class1 Generation2 standard [22], Δ*CH_j,i_*(*k*) is the frequency channel interval between R*_i_* (*CH_i_*(*k*) denotes the frequency channel numbers selected by R*_i_* at the *k*th time slot) and R*_j_* (*CH_j_*(*k*) denotes the frequency channel numbers selected by R*_j_* at the *k*th time slot) selected, and *d_j,i_* is the relative distance between R*_i_* and R*_j_*.

By Equations (1)–(6), the effective interrogation range *r_i_* (i.e., blue circle in Figure 1) of R*_i_* could be finally obtained by solving Equation (7) to ensure that the backscatter signal from target tag could be correctly recovered.

(7) $$r_{i}=min(\arg\underset{x}{\max}SINR(x,d_{j,i})\geq SINR_{min},r_{max,i})$$

According to *d_j,i_* and *r_i_* (*i, j*∈[1, *N_Reader_*]), R*_i_* may face two kinds of interference scenarios [23] at one time slot when it tries to detect the target tags:

- Multiple readers are simultaneously operating at the same frequency channel, which leads to the signals generated by multiple readers interfere with the reception system of the desired reader. In Figure 1, R_1_ suffers from this kind of interference if R_2_ and R_3_ are simultaneously operating with R_1_ at the same frequency channel. As a result, *r*_1_ is smaller than the distance *x* between R_1_ and the target tag A, R_1_ could not successfully detect A. To ensure that R_1_ could operate with a larger interrogation range to cover tag A, R_1_ with R_2_ and R_3_ should be scheduled to operate at different frequency channels and/or at different times by polling server.
- Multiple readers operating at independently frequency channels try to simultaneously detect the same tag located in the overlapped area of their effective interrogation ranges. In Figure 1, tag B locates in the overlapped interrogation area of R_1_ and R_3_, where B receives electromagnetic waves from both R_1_ and R_3_ simultaneously, it is not able to select a particular reader to communicate even if R_1_ and R_3_ are operating at the different frequency channels. To avoid this scenario, R_1_ and R_3_ should operate asynchronously which are scheduled by polling server as well.

### 2.3. The Proposed Resource Allocation Model in Centralized MRE

In order to construct the resource allocation model for a centralized MRE, it is necessary to efficiently decrease the above two interference scenarios. Accordingly, Table 1 summarizes the restrictions set for any R*_i_* and R*_j_* operating in MRE, where *d_f-min_* represents the threshold of reader-to-reader range for selecting different operating frequency channel, *d_s-min_*(Δ*CH_j,i_*(*k*)) is the threshold of reader-to-reader range for selecting different operating time slot which depends on the selected frequency channel.

Based on Table 1, two constraint conditions are proposed to restrict the resource allocation for readers operating as:

- *CH_i_*(*k*) *≠ CH_j_*(*k*) if *d_j,i_* < *d _f-min_*

Based on the relative distance *d_j,i_* between R*_j_* and R*_i_*, every R*_i_* exists a neighbor set (denoted as *IS_Ri_* = {*j*|*j*∈[1, *N_Reader_*], *d_j,i_* < *d_f-min_*} with cardinality |*IS_Ri_*| = *N_Neighbor_*(*i*), *i*∈[1, *N_Reader_*]) to mark the readers located in the range of *d_f-min_*, where R*_i_* is suffered from the first kind interference if the readers from *IS_Ri_* are simultaneously operating at the same frequency channel with R*_i_* (i.e., R_1_ and R_2_ in Figure 1). According to LIs [14], *d_f-min_* is set as 16.79 m (the transmitting signal power of reader is 1 W, and the frequency is 902–928 MHz) for any two R*_i_* and R*_j_*. Therefore, R*_i_* with any R*_j_* in its *IS_Ri_* (*j ≠ i*) should be operating at different frequency channels as: *CH_i_*(*k*) *≠ CH_j_*(*k*) if *j*∈*IS_Ri_* and *j ≠ i*, where *CH_i_*(*k*) denotes the frequency channel numbers selected by R*_i_* at the *k*th time slot.

- *ω_j_*(*k*) = 0 if *d_j,i_* ≤ *d_s-min_*(Δ*CH_j,i_*(*k*))

Further, in *d_f-min_*, another smaller distance denoted as *d_s-min_* exists, where R*_i_* is suffered from the second kind interference if readers located in *d_s-min_* are operating simultaneously even if they are at different frequency channels because they are overlapped in the effective interrogation range (i.e., R_1_ and R_3_ in Figure 1). Usually, *d_s-min_* for R*_i_* and R*_j_* is variable along with the correspondingly selected frequency channels interval Δ*CH_j,i_*(*k*), which is listed in Table 2. The calculation process is attached in Appendix A.

Therefore, R*_i_* with R*_j_* in *d_s-min_* should be operating non-simultaneously as: *ω_j_*(*k*) = 0 if *d_j,i_* ≤ *d_s-min_*(Δ*CH_j,i_*(*k*)), where *ω_j_*(*k*) denotes the 0–1 function of Equation (3).

Constricted by the constraint conditions, the resource allocation model is finally constructed as Equation (8) with the objective of maximizing the total effective interrogation range (denoted as *S*) to schedule *N_Reader_* readers operating with *N_Slot_* time slots and *N_Freq_* frequency channels. Note that *NR* = {1, 2, 3, …, *N_Reader_*}, *NS* = {1, 2, 3, …, *N_Slot_*}, *CH* = {1, 2, 3, …, *N_Freq_*}, *WS* = {(*x_i_*, *y_i_*)|*x_i_*∈[0, *L_x_*], *y_i_*∈[0, *L_y_*]} and *IS_Ri_* = {*j*|*j*∈*NR*, *d_j,i_* < *d_f-min_*}.

(8) $$\{\begin{array}{l} \;Maximize\;S=\sum_{i=1}^{N_{Reader}}\sum_{k=1}^{N_{Slot}}\omega_{i}(k)\pi (r_{i}(k){)}^{2},\\ \begin{array}{l} s.t.\begin{array}{cccc} \forall i,j\in NR, & \forall k\in NS, & CH_{i}(k),CH_{j}(k)\in CH,(x_{i},y_{j})\in WS,\Delta CH_{j,i}(k)\neq 0 & \mathrm{if} \end{array}\begin{array}{c} j\in IS_{R_{i}}, \end{array}\\ \begin{array}{cc} \;\omega_{j}(k)=0 & \begin{array}{cc} \mathrm{if} & d_{j,i}\leq d_{s-min} \end{array} \end{array}(\Delta CH_{j,i}(k)) \end{array} \end{array}$$

## 3. Geometric Distribution-Based Multiple-Reader Scheduling Optimization Algorithm Using Artificial Immune System (GD-MRSOA-AIS)

According to the proposed resource allocation model, GD-MRSOA-AIS is proposed to fairly allocate the operating resources with lower reader interference and larger effective interrogation range. In GD-MRSOA-AIS, it is composed of two parts:

- GD: Geometric distribution function (GD) combined with the fairness consideration is introduced to randomize the feasible scheduling schemes with lower interferences which satisfy the constraint conditions in Equation (8). Details are described in Section 3.1;
- AIS: Artificial immune system optimization (AIS) is to optimize these feasible scheduling schemes as the optimal scheduling scheme for readers fairly operating with larger effective interrogation range. Details are presented in Section 3.2.

### 3.1. Feasible Scheduling by Geometric Distribution Function

According to the constraint conditions in Equation (8), GD-MRSOA-AIS will first produce several feasible solutions as the feasible scheduling schemes to ensure that readers could fairly operate with low signal interference.

In centralized MRE, both the interference-reader (i.e., R_1_, R_2_ and R_3_ in Figure 1) and the isolation-reader (i.e., R_4_ in Figure 1) exist, where the isolated readers could operate at any time while the interfering readers have to be scheduled by polling server to decrease the interferences as much as possible. On the other hand, it is obvious that the isolated readers keeping on operating is thriftless since the number of deployed tags in the interrogation range is constant. Therefore, it is unfair to allocate the interfering readers and isolated readers with same opportunities to operate. To enhance the fairness, the interfering readers should gain more opportunities to operate.

To efficiently and fairly allocate the available operating time slots, a geometric probability distribution function called *Sift* [24,25] is imported to replace the typical uniform distribution. Based on the character of *Sift* distribution, it could increase the probability that one contender selects one resource in a low position, winning the contention quickly. Therefore, if R*_i_* contends for the operating time slot with large quantity of neighbor readers simultaneously, most neighbors will tend to select the last few time slots, and only the minority will contend for the first few time slots, decreasing the probability of collision in slots selection and increasing the probability that R_i_ successfully selects one of the first few time slots. On the contrary, if R*_j_* contends for the operating time slots with small quantity of neighbor readers, the probability of selecting one slot almost obeys uniform distribution.

For *Sift_Function*, the probability that the *i*th reader gains the opportunity to operate at the *k*th time slot from *N_Slot_* time slots is denoted as Equation (9). Here, *N_Neighbor_*(*i*) is the cardinality of *IS_Ri_* for R*_i_*, where *N_Neighbor_*(*i*) = 1 denotes that there is no neighbors located around R*_i_* and therefore R*_i_* is an isolated reader without any possible interference.

(9) $$p{\left(k\right)}_{i}=\frac{(1-\alpha_{i})\alpha_{i}^{N_{Slot}}}{1-\alpha_{i}^{N_{Slot}}}\alpha_{i}^{-k},\begin{array}{c} 1\leq k\leq N_{Slot}, \end{array}\begin{array}{c} 0<\alpha_{i}<1, \end{array}\alpha_{i}=N_{Neighbor}{\left(i\right)}^{-1/(N_{Slot}-1)}$$

Note that when *N_Neighbor_*(*i*) = 1, then *α_i_* = 1, and $\underset{\alpha \to 1}{\lim}p{\left(k\right)}_{i}=\;1/N_{Slot}$, becoming the uniform probability distribution. Consequently, R*_i_* with bigger *N_Neighbor_*(*i*) could easily wins the *k*th time slot as a higher *p*(*k*)*_i_* while R*_j_* with smaller *N_Neighbor_(j)* has to select the operating time slot like uniform distribution as a lower *p*(*k*)*_j_*.

By using *Sift*_*Function*, it generates several feasible scheduling schemes for readers operating with the allocated time slots and frequency channels, where the feasible scheduling schemes are restricted by the constraint conditions in Equation (8). Therefore, each feasible scheduling scheme produced by *Sift*_*Function* is actually a feasible solution for Equation (8), where the pseudocode is summarized as Algorithm 1.

**Algorithm 1:** Pseudocode for Feasible Solutions Generation by *Sift_Function**Sift_Function*1 set *i* = 1;2 while *i* ≤ *N_Reader_* do3  set $\alpha_{i}=N_{Neighbor}{\left(i\right)}^{-1/(N_{Slot}-1)}$;4  set $l_{i}=random(0,1)$;5  set *k_i_*
**=**
*N_Slot_*;6   for *z* = 1 to *N_Slot_* do7    if $p_{z}=\frac{(1-\alpha_{i})\alpha_{i}^{N_{Slot}}}{1-\alpha_{i}^{N_{Slot}}}\alpha_{i}^{-z}$ >*l_i_* then *// Contend for operating time slot*8      set *k_i_ = z;*
*// R_i_ is operating at zth time slot*9      break;10     end if11    end for12    randomize *CH_i_*(*k_i_*) from *CH*; *// Allocate a frequency channel for R_i_*13    for *j* = 1 to *N_Reader_* and *j ≠ i* do14      if *j*∈*IS_Ri_* then *//*
*Constraint Condition: *CH*_i_(k) ≠ *CH*_j_(k) if j ∈IS_Ri_*15     randomize *CH_j_*(*k_i_*) from *CH*-{*CH_i_*(*k_i_*)};16     if *d_j,i_* ≤ *d_s-min_*(|*CH_j_*(*k_i_*)-*CH_i_*(k*_i_*)|) then *//*
*ω_j_(k) = 0 if d_j,i_ ≤ d_s-min_(Δ*CH*_j,i_(k))*17        set *CH_j_*(*k_i_*) = 0;18     end if19     end if20     end for21     *i*++;22  end while

For each feasible solution of Equation (8), the total effective interrogation range might be not large enough. Therefore, this feasible scheduling scheme might be not the optimal one to maximize the global effective interrogation range. To optimize these feasible scheduling schemes, the artificial immune system optimization is introduced in the following section.

### 3.2. Optimal Scheduling by Artificial Immune System Optimization

For the produced feasible scheduling schemes, they could be optimized by using artificial immune system optimization, where each candidate antibody corresponds to one feasible solution of Equation (8) produced by *Sift_Function* and the antigen corresponds to the objective function of Equation (8) with the constraint conditions. According to this, the affinity function could be formulated as Equation (10), where the process for optimizing the feasible solutions of Equation (8) is transformed to find the maximum affinity value of Equation (10) and its corresponding optimum antibody.

(10) $$\{\begin{array}{l} Affinity(Ab)=\sum_{i=1}^{N_{Reader}}\sum_{k=1}^{N_{Slot}}\omega_{i}(k)\times (r_{i}(k){)}^{2},\\ \begin{array}{l} s.t.\begin{array}{cccc} \forall i,j\in NR, & \forall k\in NS, & CH_{i}(k),CH_{j}(k)\in CH,(x_{i},y_{j})\in WS,\Delta CH_{j,i}(k)\neq 0 & \mathrm{if} \end{array}\begin{array}{c} j\in IS_{R_{i}}, \end{array}\\ \begin{array}{cc} \;\omega_{j}(k)=0 & \begin{array}{cc} \mathrm{if} & d_{j,i}\leq d_{s-min} \end{array} \end{array}(\Delta CH_{j,i}(k)) \end{array} \end{array}$$

The proposed artificial immune system optimization is predefined to iterate *N_Gen_* generations, where it exists three phases in every iterative generation as: immune clone, immune mutation and immune suppression/recruitment. In the clone phase, each antibody is acted as a parent to be correspondingly cloned for a fixed number of *N_Clone_* children antibodies, and all children antibodies go through the mutation process but only one child antibody with the highest affinity-value among the children antibodies is selected to compare with its parent antibody. If this selected child antibody is superior to its parent antibody in affinity value, it will replace its parent antibody as a new generation. Once the affinity value of this new generation is not significantly different from that of the previous generation, the suppression will be triggered, where the *P_S_* antibodies with lowest affinity-values are suppressed. Then, the corresponding numbers of antibodies produced by *Sift_Function* are recruited. This process is repeated until *N_Gen_* iteration is met. The related symbols and their descriptions are listed in Table 3.

#### 3.2.1. Initialization

To initialize the optimizing process by using artificial immune system, *N_Ini_* antibodies (feasible solutions) are randomized by repeatedly run *Sift_Function* in *N_Ini_* times to construct the candidate antibody population set as *AB* = {*Ab*(*u*)|*u*∈[1, *N_Ini_*]}.

**Figure 2:**
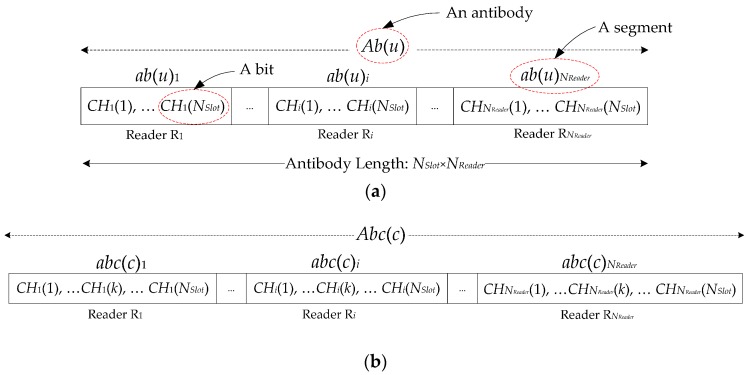
(**a**) Encoding format for the *u*th antibody individual; (**b**) Encoding format for the *c*th child antibody.

For each antibody *Ab*(*u*) in *AB*, it is encoded as integer type since both frequency channels and time slots are discrete distribution as integer type. Seen from Figure 2a, *Ab*(*u*) consists of *N_Reader_* segments as *ab*(*u*)_1_, *ab*(*u*)_2_, *ab*(*u*)_3_,…, *ab*(*u*)*_N__Reader_*, where each *ab*(*u*)*_i_* is actually corresponding to a reader R*_i_* and it includes a sequence of bits as *CH_i_*(1), *CH_i_*(2), *CH_i_*(3),…, *CH_i_*(*N_Slot_*) where each *CH_i_*(*k*) is denoted as the selected frequency channel of reader R*_i_* at the *k*th time slot. *CH_i_*(*k*) = 0 means that reader R*_i_* does not operate at this *k*th slot.

#### 3.2.2. Clone Operation

After producing the candidate antibody population set as *AB* = {*Ab*(*u*)|*u*∈[1, *N_Ini_*]}, each *Ab*(*u*) is regarded as an parent antibody. For each parent antibody *Ab*(*u*), *N_Clone_* cloned antibodies with the same encodings are produced as its children antibodies, in which these children antibodies will go through the mutation operation to increase the diversity and therefore they have more potential to evolve as excellent individuals. Here, *N_Clone_* is required to be predefined as the cloned multiplier, and each *Ab*(*u*) has a clone pool as *AB*(*u*)*_Clone_* = {*Abc*(*c*)|*c*∈[1, *N_Clone_*]} to store its children antibodies, where each *Abc*(*c*) in *AB*(*u*)*_Clone_* is encoded as Figure 2b.

Note that each child antibody *Abc*(*c*) from the clone pool *AB*(*u*)*_Clone_* is totally the same as its parent antibody *Ab*(*u*) in every bit before the mutation operation.

#### 3.2.3. Mutation Operation

For each child antibody *Abc*(*c*) (i.e., Figure 2b) from the clone pool *AB*(*u*)*_Clone_* = {*Abc*(*c*)|*c*∈[1, *N_Clone_*]} of its parent *Ab*(*u*), there are two optional mutation operators as: single-bit mutation operator is applied for the ones with higher affinity values and region-bits mutation operator is applied for the ones with lower affinity values.

For single-bit mutation operator, only one bit *CH_i_*(*k*) is randomly selected as a hotspot to be mutated following the constraint conditions, which belongs to a reader R*_i_* (i.e., Figure 2b). As a result, this mutated *Abc*(*c*) is also a feasible scheduling scheme as well.

For region-bits mutation operator, a contiguous region bits are selected to be mutated starting from a hotspot of *CH_i_*(*k*) increasing *L*-bit length, note that *L* is less than *N_Slot_* minus *k* to guarantee the mutation is happened in one reader R*_i_* (i.e., Figure 2b). In addition, this mutation is restricted by the constraint conditions. As a result, this mutated *Abc*(*c*) is also a feasible scheduling scheme.

By single-bit mutation, the children antibody could extremely inherit the superior bits of the parent antibody while the children antibody would have more potential to mutate as the superior ones by region-bits mutation. Both of them could fast the convergence and quickly reach to the global optimization.

The single-bit and region-bit mutation operator run as in Algorithms 2 and 3 respectively.

After the mutation operations, for each parent antibody *Ab*(*u*) (*u*∈[1, *N_Ini_*]), its children antibodies *Abc*(*c*) from the clone pool *AB*(*u*)*_Clone_* = {*Abc*(*c*)|*c*∈[1, *N_Clone_*]} are totally different: some *Abc*(*c*) surpasses *Ab*(*u*) in affinity value while some of *Abc*(*c*) are identical or inferior to *Ab*(*u*). To distinguish them, all *Abc*(*c*) (*c*∈[1, *N_Clone_*]) should be sorted by affinity value. Select the *Abc*(*c*) with highest affinity value and compare it with its parent antibody *Ab*(*u*): if this *Abc*(*c*) is superior to its parent antibody *Ab*(*u*), it will replace its parent antibody as a new candidate antibody in *AB*.

**Algorithm 2:** Pseudocode for Single-bit Mutation Single-bit _Operator () 1 Randomly determine a bit *CH_i_*(*k*) of R*_i_* from *Abc*(*c*) to be mutated; 2 for *j* = 1 to *N_Reader_* and *j ≠ i* 3   if *IS_Rj_*∩*IS_Ri_* ≠ ∅ 4   *CH* = *CH*-{*CH_j_*(*k*)}; 5   end if 6 end for 7 if *CH* ≠ ∅ 8   Randomize *CH_i_*(*k*) from *CH*; 9 else 10   *CH_i_*(*k*) = 0; 11 end if

**Algorithm 3:** Pseudocode for Region-bits Mutation Region-bits _Operator () 1 Randomly determine a bit *CH_i_*(*k*) of R*_i_* from *Abc*(*c*) to be mutated; 2 for(*q* = *k*; *q* ≤ *k* + *L*; *q*++) 3 for *j* = 1 to *N_Reader_* and *j ≠ i* 4 if *IS_Rj_*∩*IS_Ri_* ≠ ∅ 5 *CH* = *CH*-{*CH_j_*(*q*)}; 6 end if 7 end for 8 if *CH* ≠ ∅ 9 Randomize *CH_i_*(*q*) from *CH*; 10 else 11 *CH_i_*(*q*) = 0; 12 end if 13 end for

#### 3.2.4. Suppression Operation

After the mutation operation, the candidate antibodies are changed. If the affinity value of new candidate antibody population set *AB* is significantly increased, this *AB* will be directly regarded as a new generation and go through the same immune process via clone operation, mutation operation and suppression operation.

On the contrary, the candidate antibodies with highest affinity values should be promoted while the antibodies with lowest affinity values should be suppressed. Sorted by the affinity value, it will suppress *P_S_* percentage of antibodies with lowest affinity values. After that, *P_S_* of *N_Ini_* antibodies (feasible solutions) are produced by *Sift_Function*, where these fresh candidate antibodies are recruited in *AB* to compose a new generation population. In addition, this new generation population will go through the same immune process via clone operation, mutation operation and suppression operation.

**Figure 3:**
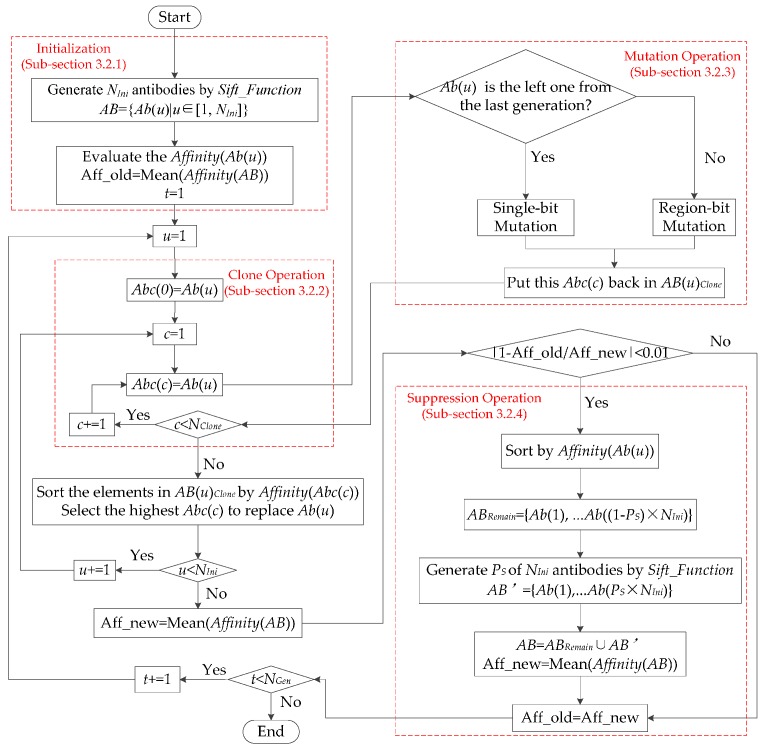
Flowchart of proposed AIS optimization in GD-MRSOA-AIS.

When the iteration reaches the predefined maximum generations *N_gen_*, GD-MRSOA-AIS terminates. Finally, the pseudocode for proposed artificial immune system (proposed-AIS) optimization in GD-MRSOA-AIS is listed as Algorithm 4, where the corresponding flowchart is shown in Figure 3.

**Algorithm 4: Pseudocode for Optimal Scheme Generation by using proposed-AIS Optimization**AIS_Optimization1  Set *N_Ini_*, *N_Clone_, N_Gen_*, *P_S_*;2  int t, u, c;3  float aff_old, aff_new;**4**  **Initialization:** Run *Sift_Function* in *N_Ini_* times to construct set AB;5       Evaluate the affinity value of all elements in AB and average them as aff_old;6  while *t*< *N_Gen_* do7   for(*u* = 1; *u* ≤ *N_Ini_*; *u*++)8    Produce *N_Clone_* clones for *u*th antibody as its children antibodies; *// Clone Operation*9    for(*c* = 1; *c* ≤ *N_Clone_*; *c*++)10      Mutate *c*th child antibody following the **Mutation Operation**;11    end for12    Evaluate the affinity value of all children antibodies and their parent antibody;13    Remain the antibody with the highest affinity value;14   Evaluate the affinity value of all candidate antibodies and average them as aff_new;15   end for16   if(aff_new is not significantly bigger than aff_old) *// Suppression Operation*17     Sort the candidate antibodies by their affinity values;18     Suppress *P_S_* antibodies with lowest affinity values;19     Produce *P_S_* new recruited antibodies by Sift_Function satisfying the constraint conditions;20   end if21 end while

## 4. Simulations and Results

In this section, the total effective interrogation range using the proposed-AIS optimization with *Sift_Function* (denoted as GD-MRSOA-AIS) and without *Sift_Function* (denoted as MRSOA-AIS) will be simulated and compared with the state-of-the-art algorithm (LIs [14]) in two scenarios:

- The number of readers *N_Reader_* = 15 with fixed positions among GD-MRSOA-AIS, MRSOA-AIS and LIs. Meanwhile, the advantage of *Sift_Function* and proposed-AIS optimization is illustrated by comparisons of GD-MRSOA-AIS, MRSOA-AIS and LIs.
- Variable *N_Reader_* with randomized positions among GD-MRSOA-AIS, MRSOA-AIS and LIs.

During the simulations, readers are randomly distributed over an area in two dimensions with 100 × 100 m^2^ (i.e., *L_x_* = 100 m, *L_y_* = 100 m). As readers communicate with tags by means of EPCGlobal C1G2, the parameter settings are same with the ones listed in Table A1 in Appendix A.

Given a certain number of readers, as the number of available time slots increases, the readers have more options to avoid simultaneously operation. In other words, for a specific number of time slots, as the number of readers increases, the total effective interrogation range will be smaller, which is caused by the heavy interferences. Without the loss of generality, *N_Slot_* is finally assigned as 5 in the following simulations. In addition, as the number of available frequency channels increases, the total effective interrogation range always increases since each reader has more options for selecting an available frequency channel to simultaneously operate with other readers at different frequency channels. Usually, the number of available frequency channels is set to no more than 10. Consequently, *N_Freq_* is assigned as 10 in the simulation. The parameters setting of proposed-AIS optimization in simulation are listed in Table 4.

### 4.1. Case Study: N_Reader_ = 15, N_Slot_ = 5, N_Freq_ = 10

**Figure 4:**
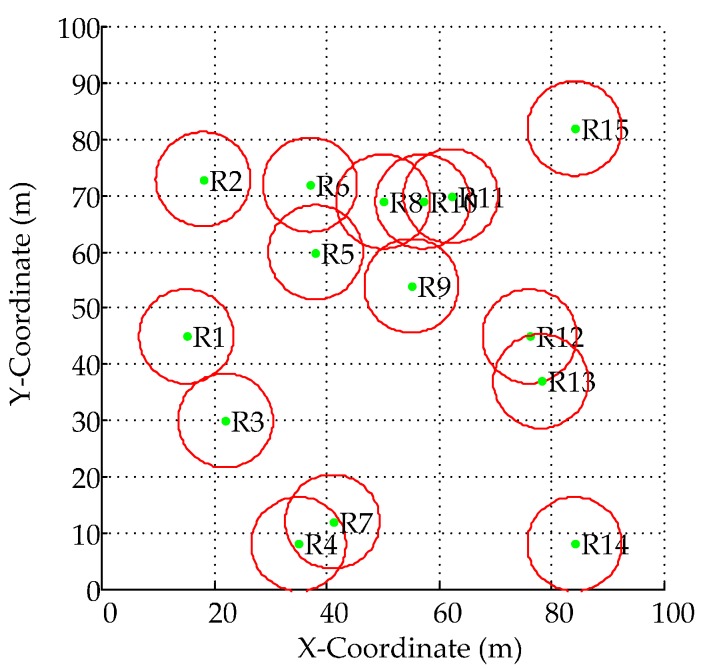
Fixed Location of Readers (*N_Reader_* = 15).

When *N_Reader_* = 15, the fixed positions of readers are randomized in Figure 4, where the corresponding neighbor set for each reader is *IS*_R1_ = {1, 3}, *IS*_R2_ = {2, 5, 6}, *IS*_R3_ = {1, 3}, *IS*_R4_ = {4, 7}, *IS*_R5_ = {5, 6, 8, 9}, *IS*_R6_ = {2, 5, 6, 8}, *IS*_R7_ = {4, 7}, *IS*_R8_ = {5, 6, 8, 9, 10, 11}, *IS*_R9_ = {5, 8, 9, 10, 11}, *IS*_R10_ = {8, 9, 10, 11}, *IS*_R11_ = {8, 9, 10, 11}, *IS*_R12_ = {12, 13}, *IS*_R13_ = {12, 13}, *IS*_R14_ = {14}, and *IS*_R15_ = {15}. Here, R_14_ and R_15_ are isolated readers since *IS*_R14_∩*IS*_R*j*_ = ∅ (any *j*∈*NR* and *j* ≠ 14) and *IS*_R15_∩*IS*_R*l*_ = ∅ (any *l*∈*NR* and *l* ≠ 15). On the contrary, i.e., *IS*_R5_∩*IS*_R8_∩*IS*_R9_∩*IS*_R10_∩*IS*_R11_ = {5, 8, 9, 10, 11}, these readers should be scheduled to operate at different time slots or simultaneously operate but at different frequency channels.

**Figure 5:**
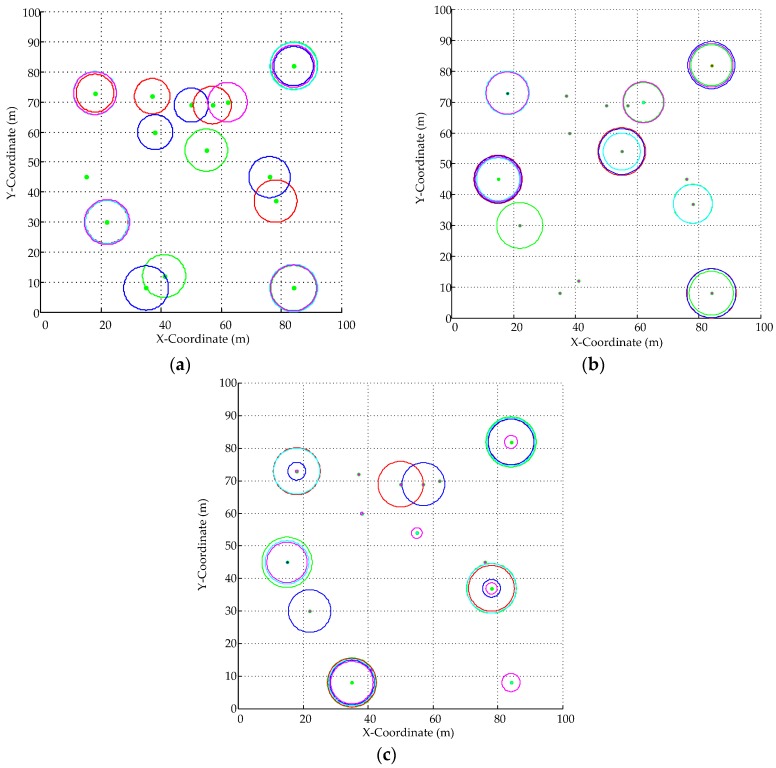
Graphical Representation of Optimal Operating Schedule for 15 readers with *N_Slot_* = 5 and *N_Freq_* = 10: (**a**) GD-MRSOA-AIS; (**b**) MRSOA-AIS; and (**c**) LIs.

Figure 5 shows the graphical representation of effective interrogation range for each reader with its corresponding operation time slots (where the five time slots are represented by Green, Red, Blue, Cyan and Magenta) under the operating scheduling scheme. Correspondingly, the operating scheduling scheme for each algorithm is listed in Table 5, where *CH_i_*(*k*) = 0 means that the *i*th reader does not operate at this *k*th time slot. At the first time slot (TS_1_) as green circles in Figure 5a–c, {R_7_, R_9_, R_14_, R_15_} are operating at the frequency channel of {*CH*_7_(1) = 5, *CH*_9_(1) = 7, *CH*_14_(1) = 1, *CH*_15_(1) = 10} using GD-MRSOA-AIS as in Table 5; {R_11_, R_13_, R_14_, R_15_} are operating at the frequency channel of {*CH*_11_(1) = 9, *CH*_13_(1) = 7, *CH*_14_(1) = 1, *CH*_15_(1) = 5} using MRSOA-AIS; and {R_1_, R_4_, R_13_, R_15_} are operating at the frequency channel of {*CH*_1_(1) = 7, *CH*_4_(1) = 1, *CH*_13_(1) = 10, *CH*_15_(1) = 3} using LIs algorithm. Take {R_4_, R_7_} as an example, *IS*_R4_∩*IS*_R7_ = {4, 7}. According to Table 5, R_4_ is scheduled to operate at TS_3_ with the frequency channel of *CH*_4_(3) = 10 and R_7_ is scheduled to operate at TS_1_ with the frequency channel of *CH*_7_(1) = 5 by GD-MRSOA-AIS while R_4_ is scheduled to operate at every time slot as {*CH*_4_(1) = 1, *CH*_4_(2) = 3, *CH*_4_(3) = 1, *CH*_4_(4) = 1, *CH*_4_(5) = 3} and R_7_ is scheduled to be inactive at every time slot by LIs. Therefore in LIs, R_4_ is actually operating in uselessness especially for the last few time slots and it is unfair for R_7_. On the contrary, GD-MRSOA-AIS schedules R_4_ and R_7_ corporately operating in a fair way. The same scenario is also happened in {R_12_, R_13_}.

On average, the effective interrogation radius using GD-MRSOA-AIS is 2.142 m, 2.0881 m using MRSOA-AIS and 1.909 m using LIs algorithm. It seems like that the effective interrogation ranges of these three algorithms are similar. However, by introducing *Sift_Function*, GD-MRSOA-AIS is obviously fairer since almost all readers (except for R_1_) are scheduled to operate at different time slots and frequency channels while both of MRSOA-AIS and LIs could not. For example, {R_4_, R_5_, R_6_, R_7_, R_8_, R_10_, R_12_} are always inactive in MRSOA-AIS and {R_6_, R_7_, R_11_, R_12_} are inactive in LIs (shown in Table 5 and Figure 5b,c), especially that R_6_, R_10_, R_11_ are posited in crowed with more neighbors (shown in Figure 4).

To evaluate the fairness of readers during the operating scheduling, a general purpose quantitative measure of fairness called Jain Index [26] is imported as Equation (11), where *N_Reader_* denotes the number of readers, *ω_i_* is the 0–1 function to indicate whether the reader R*_i_* operates at least once at these *N_Slot_* time slots or not, and *N_Neighbor_*(*i*) is the cardinality of *IS_Ri_*. The *I_Jain_* ranges from 0 to 1: the higher *I_Jain_* means that the corresponding optimal scheduling scheme is fairer. Obviously, R*_i_* with larger *N_Neighbor_*(*i*) will make more contribution to obtain a bigger *I_Jain_*. Therefore, the Jain Index could express the fairness of an optimal scheduling scheme.

(11) $$I_{Jain}=\frac{\sum_{i=1}^{N_{Reader}}\omega_{i}\times N_{Neighbor}(i)}{N_{Reader}\times N_{Reader}}$$

As a result, GD-MRSOA-AIS becomes the fairest one with highest Jain Index of *I_Jain_* = 18.22%, while the *I_Jain_* of MRSOA-AIS is 8.89% and the *I_Jain_* of LIs is 14.67% in the same scenario.

**Figure 6:**
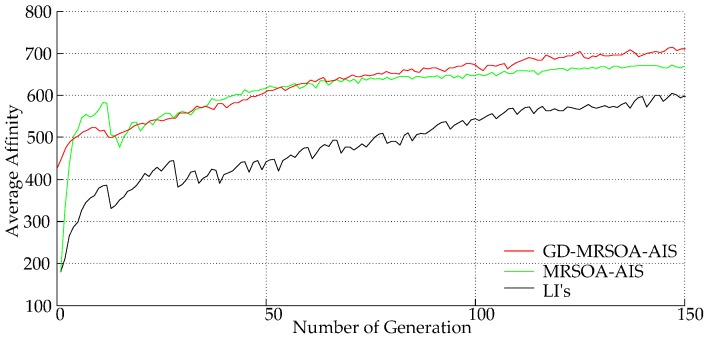
Affinity value versus number of generations.

In Figure 6, the initial average affinity value of GD-MRSOA-AIS starts from 450, which is much higher than that of the other two from 200. This obvious difference is owing to the contending mechanism of *Sift_Function* for time slots selection. Additionally, the average affinity value of both GD-MRSOA-AIS and MRSOA-AIS are much higher than LIs, where MRSOA-AIS rockets up to a high affinity value and then it stabilizes in the similar average affinity value like GD-MRSOA-AIS, which dues to the multiple mutation operations in the proposed-AIS. To achieve the average affinity value of 600, with GD-MRSOA-AIS and MRSOA-AIS, it costs around 50 generations while with LIs it is more than 150 generations. Therefore, the proposed-AIS also reduces the time consumption of optimization process.

### 4.2. Variable N_Reader_ with Fixed N_Slot_ = 5 and N_Freq_ = 10 in Randomized Positions

For a given *N_Reader_* readers with *N_Slot_* = 5 and *N_Freq_* = 10 in the area of 100 × 100 m^2^, the position of each reader is randomly located. To ensure the convergence of the results, all the simulations are repeated for 50 times with 50 randomly positions. For an easier understanding, the results are listed in five indices (the best, the worst, and the mean of total effective interrogation area, the average radius and Jain Index) among GD-MRSOA-AIS, MRSOA-AIS and LIs algorithm, which are represented in Table 6.

When *N_Reader_* = 4 with *N_Slot_* = 5 available time slots and *N_Freq_* = 10 frequency channels, readers are easily scheduled to operate at different time slots and frequency channels. Additionally, they could be deployed so sparsely that they are hardly interfered each other in such a large zone (100 × 100 m^2^). Therefore, the Jain Index is failed to measure the fairness. However, limited by the self-rated-power, the interrogation range is also finite. As a result, the total effective interrogation range is not large enough. As the number of readers increases, such as *N_Reader_* = 24 and *N_Reader_* = 28, positions of readers become much denser, the readers could not make full use of the frequency channels and the time slots, and then the individual effective interrogation range is declining and the total effective interrogation range relies on the cooperation among readers operating.

From Table 6, we can see that the Jain Index is fluctuated up and down. For the former situations (i.e., *N_Reader_* = 8, *N_Reader_* = 12, *N_Reader_* = 16), each reader has fewer neighbors since they are sparsely distributed, which declines the Jain Index value even if all of the readers are scheduled to operate at least once. For the latter situations (i.e., *N_Reader_* = 24, *N_Reader_* = 28), readers are located in denser positions and so each reader has more neighbors, but some readers might be not scheduled to operate even once (*ω_i_* = 0) which also decreases the Jain Index value. Even so, the Jain Index value of GD-MRSOA-AIS is also higher than MRSOA-AIS and LIs with the same number of readers.

In addition, Table 6 shows that the best, the worst and the mean of total effective interrogation range between GD-MRSOA-AIS and MRSOA-AIS are similar but larger than the ones of LIs; meanwhile, the average radius of effective interrogation range for each operating readers with GD-MRSOA-AIS and MRSOA-AIS is also larger than that with LIs. The total effective interrogation range is declined fast by using LIs when the number of readers increases while it is much slower by using both GD-MRSOA-AIS and MRSOA-AIS. For example, when *N_Reader_* = 20, the mean effective interrogation range with GD-MRSOA-AIS is 3133.686 m^2^, the best one as 3331.845 m^2^ and the worst one as 2935.414 m^2^; the mean value with LIs is only 2528.864 m^2^, the best one as 2869.225 m^2^ and the worst one as 2297.590 m^2^. When *N_Reader_* = 28, with GD-MRSOA-AIS, the mean value is glided down to 2737.177 m^2^, the best one as 3068.395 m^2^ and the worst one as 2436.688 m^2^; with LIs, the mean value is slumped down as 1670.742 m^2^, the best one as 1859.409 m^2^ and the worst one as 1532.377 m^2^.

In conclusion, GD-MRSOA-AIS could schedule multiple readers operating in fairness with lower multiple-reader interfering and large effective interrogation range, where the fairness with lower multiple-reader interfering dues to the geometric distribution of *Sift_Function* and the large effective interrogation range dues to the proposed-AIS optimization process. As a result, the deployment of eight readers with five available time slots and 10 available frequency channels is the most economical way to maximize the total effective interrogation range in 100 × 100 m^2^, especially using GD-MRSOA-AIS.

## 5. Conclusions

This paper refers to the multiple-reader interference problem in the multiple-reader environment (MRE) and formulates a resource allocation model with the consideration of interference avoidance in SINR under EPCGlobal C1G2 standard, where the allocable resources include available time slots and frequency channels. Based on this resource allocation model, GD-MRSOA-AIS is further proposed. In GD-MRSOA-AIS, geometric distribution replaces the uniform distribution to produce the feasible scheduling schemes, where the reader, which is interfered by large quantity of readers, has more opportunities to be allocated in an operating time slot. To optimize these feasible scheduling schemes, the artificial immune system optimization including immune clone, immune mutation and immune suppression is further introduced. During the mutation operation for antibodies, there are two optional mutation operators depending on the affinity values. According to the simulation results, GD-MRSOA-AIS could obtain an optimal and fairer readers collaboratively operating scheduling scheme, and it could output a larger total effective interrogation range with higher convergence than the state-of-the-art one.

Although GD-MRSOA-AIS indeed enhances the effective interrogation range with lower interferences by fairer operating scheme, it still has shortcomings:

- It only focuses on the scenario of multiple readers in known position with randomized tags in fixed positions, and ignores the new entrancing tags with variable population.
- It only solves the resource allocation problem for multiple readers scheduling to interrogate the tags in centralized MRE, but ignores the identification problem (i.e., tag-to-tag collision) in data transmission process.

Therefore, the next work will reconstruct a resource allocation model by considering the variable population of tags and identification problem with an enhanced scheduling-based algorithm.

## Figures and Tables

**Table 1:** Readers operating restrictions versus *d_j,i_*.

	=Frequency Channel	≠Frequency Channel
=time slot	*d_j,i_* > *d_f-min_*	*d_f-min_ ≥ d_j,i_* > *d_s-min_*(Δ*CH_j,i_*(*k*))
≠time slot	*d_s-min_*(Δ*CH_j,i_*(*k*)) *≥ d_j,i_* > 0	*d_s-min_*(Δ*CH_j,i_*(*k*))*≥ d_j,i_* > 0

**Table 2:** Influence of frequency channel interval to *d_s-min_*.

Δ*CH_j,i_*(*k*)	0	1	2	3	≥4
***d_s-min_*(Δ*CH_j,i_*(*k*))**	0.0269 m	0.8452 m	2.6970 m	6.7458 m	10.339 m

**Table 3:** Symbol and description in artificial immune system optimization.

Symbol	Description
*AB*	The candidate antibody population
*Ab*(*u*)	The *u*th antibody
*ab*(*u*)*_i_*	The *i*th antibody segment in *Ab*(*u*)
*AB_Remain_*	The set for remained antibodies
*Ab*(*u*)*_Clone_*	The clone pool for *Ab*(*u*)
*Affinity*(*·*)	Affinity function
*Aff_old*	The mean affinity value of the previous generation
*Aff_new*	The mean affinity value of the current generation
*CH_i_*(*k*)	The *k*th bit in *ab(u)_i_*
*N_Ini_*	The population size of *AB*
*N_Clone_*	The cloned multiplier
*N_Gen_*	The generation multiplier
*L*	Region-bits mutation length
*P_S_*	The suppression percentage

**Table 4:** Parameter settings of proposed AIS optimization process.

Parameter	Value
The number of available time slots (*N_Slot_*)	5
The number of available frequency channels (*N_Freq_*)	10
Initialized candidate population size (*N_Ini_*)	40
Cloned Multiplier (*N_Clone_*)	5
Maximum generation (*N_Gen_*)	150
Suppression percentage (*P_S_*)	25%

**Table 5:** The operating schedule for 15 readers with *N_Slot_* = 5 and *N_Freq_* = 10 among three algorithms.

			GD-MRSOA-AIS					MRSOA-AIS					LIs				
		TS_*k*_	TS_1_	TS_2_	TS_3_	TS_4_	TS_5_	TS_1_	TS_2_	TS_3_	TS_4_	TS_5_	TS_1_	TS_2_	TS_3_	TS_4_	TS_5_
	*CH*_*i*_(*k*)																
R_*i*_																	
R_1_			0	0	0	0	0	0	**6**	**7**	**1**	**1**	**7**	0	0	**3**	**5**
R_2_			0	**1**	0	**7**	**10**	0	0	0	**10**	**7**	0	**1**	**8**	**7**	**1**
R_3_			0	**10**	0	**5**	**1**	0	**3**	0	0	0	0	0	**5**	0	0
R_4_			0	0	**10**	0	0	0	0	0	0	0	**1**	**3**	**1**	**1**	**3**
R_5_			0	0	**1**	0	0	0	0	0	0	0	0	0	0	0	**1**
R_6_			0	**5**	0	0	0	0	0	0	0	0	0	0	0	0	0
R_7_			**5**	0	0	0	0	0	0	0	0	0	0	0	0	0	0
R_8_			0	0	**5**	0	0	0	0	0	0	0	0	**10**	0	0	0
R_9_			**7**	0	0	0	0	0	**2**	**10**	**5**	0	0	0	0	0	**8**
R_10_			0	**8**	0	0	0	0	0	0	0	0	0	0	**3**	0	0
R_11_			0	0	0	0	**8**	**9**	0	0	0	**5**	0	0	0	0	0
R_12_			0	0	**8**	0	0	0	0	0	0	0	0	0	0	0	0
R_13_			0	**3**	0	0	0	**7**	0	0	**3**	0	**10**	**7**	**7**	**10**	**7**
R_14_			**1**	0	0	**9**	**6**	**1**	**9**	**2**	0	0	0	0	0	0	**10**
R_15_			**10**	0	**3**	**3**	**3**	**5**	0	**5**	**7**	**10**	**3**	**5**	**10**	**5**	**9**

* In the table, TS*_k_* stands for the *k*th time slot from the optional values of {TS_1_, TS_2_, TS_3_, TS_4_, TS_5_} in *N_Slot_* = 5. R*_i_* stands for the *i*th reader from the optional values of {R_1_, R_2_, …, R_15_} in *N_Reader_* = 15. The operating frequency channel for *i*th reader at *k*th time slot (*CH_i_*(*k*)) is listed from the optional values of *CH* = {0, 1, 2, 3, …, 10} when *N_Freq_* = 10, where *CH_i_*(*k*) = 0 means that the *i*th reader does not work at *k*th time slot.

**Table 6:** The comparison results of the total effective interrogation range with *N_Slot_* = 5 and *N_Freq_* = 10.

*N_Reader_*	Algorithm	Best (Unit: m^2^)	Worst (Unit: m^2^)	Mean (Unit: m^2^)	Average Radius (Unit: m)	Jain Index (*I_Jain_*)
4	GD-MRSOA-AIS	3886.166	2459.977 *	3190.954 *	6.906 *	**-**
	MRSOA-AIS	3976.631 *	1918.756	3078.580	6.784	**-**
	LIs	3570.661	1512.397	3041.527	6.573	**-**
8	GD-MRSOA-AIS	4392.362 *	3287.613 *	3907.504 *	4.308 *	42.19% *
	MRSOA-AIS	4293.650	3180.301	3790.565	4.174	25.00%
	LIs	4316.239	3263.520	3859.630	4.252	29.69%
12	GD-MRSOA-AIS	3910.000 *	3328.207 *	3539.014 *	2.670 *	27.08% *
	MRSOA-AIS	3765.571	3014.043	3481.625	2.620	13.89%
	LIs	3671.977	2977.352	3428.592	2.639	18.06%
16	GD-MRSOA-AIS	3780.408 *	3356.667 *	3533.103 *	2.027 *	27.34% *
	MRSOA-AIS	3612.826	2901.954	3471.431	1.974	19.92%
	LIs	3437.009	3128.828	3270.978	1.963	21.88%
20	GD-MRSOA-AIS	3331.845	2935.414 *	3133.686	1.443	70.50% *
	MRSOA-AIS	3369.615 *	2901.954	3160.941 *	1.463 *	40.25%
	LIs	2869.225	2297.590	2528.864	1.346	45.50%
24	GD-MRSOA-AIS	3232.966 *	2550.474 *	2909.812 *	1.229 *	68.06% *
	MRSOA-AIS	3206.098	2279.184	2830.733	1.195	43.75%
	LIs	2151.093	1874.913	2035.651	1.020	42.36%
28	GD-MRSOA-AIS	3068.395 *	2436.688	2737.177 *	0.999 *	64.29% *
	MRSOA-AIS	2675.789	2586.912 *	2616.538	0.923	35.71%
	LIs	1859.409	1532.377	1670.742	0.814	32.14%

* The best one for each comparison.

## Appendix A

Assume that readers with the rated power of 30 dB m (1 W) are operating at the frequency of 902–928 MHz with the bandwidth of 500 KHz and noise power of −90 dB m according to EPCGlobal C1G2 [22]. The corresponding parameters are listed in Table A1.

**Table A1:** Parameter value under EPCGlobal C1G2 standard.

Parameter	Description		Value
*E_tag_*	The effective power reflection coefficient of the tag		0.1
*α_bw_*	The normalized spectrum power		0.86
*γ*	The path loss exponent		2.5
*M_d_*	The modulation depth		0.1
*H*	The fading coefficient in the channel between R*_i_* and R*_j_*		1
*P_min_*	The minimum required power for tag operation		−15 dB m
*P_i_*	The signal power of R*_i_*		30 dB m
*P*_0_	The referenced path loss at the distance of 1 m		−31.6 dB m
*No_i_*	The noise power of *R_i_*		−90 dB m
*SINR_min_*	The minimum *SINR*		11.6 dB
*G_T_*	The reader antenna transmitting gain		6 dB
*G_R_*	The reader antenna receiving gain		6 dB
*β_mask_*(·) for MRE	The function of the spectrum mask in multiple-reader environment	Δ*CH_j,i_*(*k*) = 0	0 dB
		Δ*CH_j,i_*(*k*) = 1	−20 dB
		Δ*CH_j,i_*(*k*) = 2	−50 dB
		Δ*CH_j,i_*(*k*) = 3	−60 dB
		Δ*CH_j,i_*(*k*) ≥ 4	−65 dB

For any R*_i_* and R*_j_*, the effective interrogation range might be overlapped if the distance between these two readers is close enough. For this scenario, readers are not preferred to operate simultaneously even though they are at different frequency channels. To determine this boundary distance, it is derived as follows.

$$\begin{array}{l} According\begin{array}{c} to\begin{array}{c} Equation(7), \end{array} \end{array}\\ r_{i}={\left(\frac{BP}{I_{j}\times \beta_{mask}(\Delta CH_{j,i}(k))\times d_{j,i}^{-\gamma }+SN_{i}}\right)}^{\frac{1}{2\gamma }},\\ \begin{array}{c} where \end{array}BP=\alpha_{bw}E_{tag}P_{i}G_{T}G_{R}P_{0}{}^{2},I_{j}=SINR_{\min}hP_{j}G_{T}G_{R}P_{0},SN_{i}=SINR_{\min}\times No_{i},\\ Suppose\begin{array}{c} that\begin{array}{c} r_{i} \end{array} \end{array}\leq \frac{d_{j,i}}{2},\\ \Rightarrow \\ {\left(\frac{BP}{I_{j}\times \beta_{mask}(\Delta CH_{j,i}(k))\times d_{j,i}^{-\gamma }+SN_{i}}\right)}^{\frac{1}{2\gamma }}\leq \frac{d_{j,i}}{2},\\ \Rightarrow \\ \begin{array}{c} Suppose \end{array}\begin{array}{c} that \end{array}A=I_{j}\times \beta_{mask}(\Delta CH_{j,i}(k)),\begin{array}{c} B=SN_{i} \end{array},\\ \begin{array}{c} then \end{array}{\left(\frac{BP}{A\times d_{j,i}^{-\gamma }+B}\right)}^{\frac{1}{2\gamma }}\leq \frac{d_{j,i}}{2},\\ \Rightarrow \\ Suppose\begin{array}{c} that\begin{array}{c} f(d_{j,i})= \end{array}A \end{array}\times d_{j,i}^{\gamma }+B\times d_{j,i}^{2\gamma }-4^{\gamma }\times BP,\\ and\begin{array}{c} let\begin{array}{c} x= \end{array} \end{array}d_{j,i}^{\gamma },\\ then\begin{array}{c} f(x)=Ax+Bx^{2} \end{array}-4^{\gamma }BP,\\ \Rightarrow \\ \Delta =A^{2}+4\times B\times 4^{\gamma }BP>0,\\ x=\frac{-A\pm \sqrt{\Delta }}{2B},\\ let\begin{array}{c} x\geq 0 \end{array}\\ \Rightarrow \\ d_{j,i}=\sqrt[{\gamma }]{x}\\ \Rightarrow \\ According\begin{array}{c} to\begin{array}{c} \beta_{mask}(\cdot ) \end{array} \end{array}\begin{array}{c} in\begin{array}{c} MRE, \end{array} \end{array}\\ when\begin{array}{c} \Delta CH_{j,i}(k) \end{array}=0,\begin{array}{c} d_{j,i}=0.0269m \end{array},\\ when\begin{array}{c} \Delta CH_{j,i}(k) \end{array}=1,\begin{array}{c} d_{j,i}=0.8452m \end{array},\\ when\begin{array}{c} \Delta CH_{j,i}(k) \end{array}=2,\begin{array}{c} d_{j,i}=2.6970m \end{array},\\ when\begin{array}{c} \Delta CH_{j,i}(k) \end{array}=3,\begin{array}{c} d_{j,i}=6.7458m \end{array},\\ when\begin{array}{c} \Delta CH_{j,i}(k) \end{array}\geq 4,\begin{array}{c} d_{j,i}=10.3390m \end{array}. \end{array}$$
